# Biodegradable Electrospun Nanofiber Membranes as Promising Candidates for the Development of Face Masks

**DOI:** 10.3390/ijerph20021306

**Published:** 2023-01-11

**Authors:** Rujun Shen, Yunlong Guo, Shuaijie Wang, Ayikezi Tuerxun, Jiaqi He, Ye Bian

**Affiliations:** Key Laboratory of Energy Thermal Conversion and Control of Ministry of Education, School of Energy and Environment, Southeast University, Nanjing 210096, China

**Keywords:** electrospinning, nanofiber, face mask, biodegradability

## Abstract

Aerosol particles, such as the widespread COVID-19 recently, have posed a great threat to humans. Combat experience has proven that masks can protect against viruses; however, the epidemic in recent years has caused serious environmental pollution from plastic medical supplies, especially masks. Degradable filters are promising candidates to alleviate this problem. Degradable nanofiber filters, which are developed by the electrospinning technique, can achieve superior filtration performance. This review focuses on the basic introduction to air filtration, the general aspects of face masks, and nanofibers. Furthermore, the progress of the state of art degradable electrospun nanofiber filters have been summarized, such as silk fibroin (SF), polylactic acid (PLA), chitosan, cellulose, and zein. Finally, the challenges and future development are highlighted.

## 1. Introduction

Infectious diseases have brought great disasters to humans for thousands of years, especially the respiratory infections caused by microorganisms, such as tubercle bacillus, SARS coronavirus, and COVID-19, which is highly contagious and has spread worldwide, posing ongoing global concerns. It is reported that hundreds of millions of people were infected by COVID-19, resulting in millions of deaths, according to World Health Organization (WHO).

Since the novel coronavirus can be carried by droplets, gases, aerosols, and contact, the most effective protection to cut off the transmission is personal protective equipment (PPE), including face masks, gloves, gowns, aprons, hoods, eye-shields, and shoe covers [1,2,3,4]. Recommended by WHO to reduce the spread of disease, face masks account for a large proportion of the consumption of PPE, especially single-use face masks (SFMs).

The filter layer of conventional SFM is mainly undegradable petro-based polypropylene melt-blown cloth, capturing ultrafine particles through electrostatic adsorption. Therefore, the filtration efficiency decreases continuously because of the humidity change caused by the moisture breathed out by wearers. Due to the lack of durability and the incredible increase in consumption, the undegradable discarded masks become substantial plastic waste.

It is estimated that about 3.4 billion SFMs are discarded every day during the pandemic worldwide [5]. These plastic wastes pollute the land and pose a threat to the marine environment. Moreover, it is found that facemasks are possible sources of emerging pollutants, which release microplastic fibers into the water [6], aggravating global plastic pollution.

Nanofibrous membrane technology has emerged as a promising alternative to energy-intensive separation processes [7,8,9,10,11,12]. The porous structure with large specific surface areas provides a long-lasting filtration efficacy through physical sieving and is much more energy-efficient, compared to electrostatic adsorption. The particular structural features of nanofibers also lay the foundation for the development of multi-functional membranes used as facemasks, such as nanofibrous membranes with high interception efficiency and antimicrobial property [13].

Based on membrane technology, biodegradable filtration media is a possible solution to serious plastic pollution. Therefore, there is an urgent need to move away from petrochemical-based raw materials and towards greener bio-based resources. Bioderived materials have been widely studied and developed into nanofibers [14], including chitosan [15], β-Cyclodextrin [16], collagen [17], soy protein [18], etc.

As a sort of popular materials, various polymers have a wide range of applications. For example, porous organic polymers can be applied for water purification and sewage treatment [19]. These studies shed light on the possible applications of bio-based nanofiber materials. Moreover, due to their biocompatibility and biodegradability, bio-based fiber materials have more potential to be utilized in the biomedical industry, consisting of drug delivery [20,21], tissue engineering [22], etc.

## 2. Basic Introductions to Air Filtration

### 2.1. Filtration Mechanism

Filtration can be defined as the process of separating solid particles from a polluted air environment, thus improving the purity of the coming airflow [23,24,25,26]. When a particle is captured by the filter, there exist several capture mechanisms, such as inertia, diffusion, interception, gravity, and electrostatic deposition [27].

(1) Interception effect. When a particle flows with the air streamline, it can be captured by the fiber if the distance between the fiber and particle is within one particle radius [28]. Commonly, a strong interception effect prefers the particle with a large size [29].
(2) Gravity effect. The particles are affected by gravity and fail to deposit on the surface of the filter material with the gas through the pores [28]. As the mass increases, the effect of gravity efficiency will enhance [29].
(3) Inertia effect. The inertia effect refers to a particle that cannot move along the original path, but is deposited on the fiber due to its inertia [28]. Therefore, the inertia effect would be much more obvious for a fast-moving aerosol particle [30]. Moreover, the strong inertia effect prefers a particle with a large size [31].
(4) Diffusion effect. When tiny particles move along the streamline near the fiber, the irregular Brownian motion can enhance these particles to hit the fiber [28]. The particle diffusion distance can reach dozens of times larger than the fiber spacing, so the particles contact the fiber surface and deposition [32]. The diffusion effect is mainly reflected in the particles with small sizes. Moreover, low gas velocity can also result in predominant diffusion.
(5) Electrostatic effect. The electrostatic effect can greatly improve filtration efficiency; however, the electrostatic effect is often neglected, unless the fibers or particles have been charged [26,33]. A neutral particle can be attracted to a charged fiber, and a charged particle can also be attracted to a neutral fiber. Moreover, because of the coulombic attraction, the electrostatic effect can work when the fiber and particle are oppositely charged.

**Figure 1:**
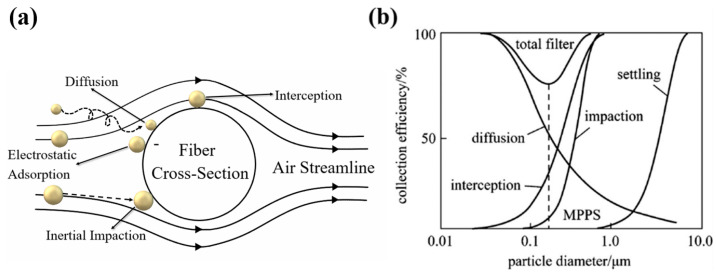
(**a**) Schematic showing of filtration mechanisms. (**b**) Collection efficiency of particles with different diameters [35].

The above mechanisms can result in the capture of particles by the filter. Additionally, superior filtration performance comes along with the effect of multi-mechanisms [34]. Figure 1a illustrates the filtration mechanisms of particles. Generally, particles in larger sizes prefer to be captured by inertia and the interception effect. Small particles, especially those below 100 nm, are easily captured by the diffusion effect. The collection efficiency of particles with different diameters is shown in Figure 1b.

### 2.2. Filtration Performance Index

There are three main indicators to evaluate the filtration performance of filter materials, which are filtration efficiency, pressure drop, and quality factor (QF) [36].

#### 2.2.1. Filtration Efficiency

Filtration efficiency is an important index to evaluate the performance of a filter, which reflects the filtration capacity of filter materials [37]. It directly reflects the changes in particle concentration in the airflow before and after filtration, which can be calculated with the following equation:

$$\eta =\left(1-\frac{N_{2}}{N_{1}}\right)\times 100\%$$

where η is the filtration efficiency; $N_{1}\;$(mg/m^3^) and $N_{2}$ (mg/m^3^) refer to the particle concentration before and after filtration, respectively.

#### 2.2.2. Pressure Drop

Pressure drop refers to the difference between the pressure before and after the air flow passes through the filter material. For the mask membrane, the pressure drop is an important index for evaluating the wearing comfort, which can be defined as:

$$∆P=P_{2}-P_{1}$$

where ∆P is the pressure drop; $P_{2}$ (Pa) and $P_{1}$ (Pa) refer to the pressure before and after filtration, respectively.

#### 2.2.3. Quality Factor

For filters, the improvement of filtration efficiency will commonly increase the pressure drop. Similarly, the reduction of pressure drop will sacrifice filtration efficiency. Therefore, only considering the filtration efficiency or pressure drop cannot make a comprehensive evaluation of the filter [38]. The quality factor (QF) is an index that can take both the filtration efficiency and pressure drop into account to characterize the overall filtration performance of a filter, which can be calculated using the following equation:

$$QF=-\frac{ln\left(1-\eta \right)}{\Delta P}$$

where QF is the quality factor; η is the filtration efficiency; ΔP is the pressure drop.

The higher the filtration efficiency and the lower the pressure drop of the filter material, the greater the value of the quality factor for a filter.

In addition, the stability, dust capacity, service life, and degradation time of filter materials can also be included in the standards of evaluating the performance of filter materials [39].

## 3. General Aspects of Face Masks

The face mask has been one of the promising candidates for cutting down on the spread of COVID-19 through respiratory droplets. There are three most commonly used face masks for preventing disease transmission, which are cloth masks, surgical masks, and respirator masks. The introduction to those face masks is discussed in the following sections.

### 3.1. Cloth Mask

Cloth masks often have two layers and are made of fabrics, including cotton, silk [40], etc. The filtration efficiencies of single cloth layers range from 5% to 95% for different sizes of particles, and the combination of different fabrics exhibited a higher efficiency at 80% (for particles >300 nm) [41]. It is proven that cloth masks are effective at stopping airborne transmission [42]. Though wearing cloth face masks in public effectively slows the spread of respiratory transmitted diseases and viruses, including SARS-CoV-2 [43], it is less efficacious, compared to surgical masks.

### 3.2. Surgical Mask

Surgical masks are commonly used to protect the wearers from splashes, pathogenic bacteria or sprays of blood, or other forms of body fluids. The masks are typically three layers with two nonwoven fabric layers and a middle layer of melt-blown polymer fabric for filtration [44]. The outer layer is usually water-resistant, while the inner layer is water-absorbing. The polymers commonly used are polyethylene (PE), polypropylene (PP), polyamide (PA), and polyethylene terephthalate (PET) [45]. Surgical masks utilize electrostatic interaction for effective particle capture [46,47], which may also result in the decline of filtration efficiency, caused by the humidity change.

### 3.3. Respirator Mask

The significant difference between surgical masks and respirators (N95, FFP2, and FFP3) is that respirators are well-designed to protect users from respiratory infections, due to their good sealing properties, which contribute to the significantly higher filtration rate. Different types of respirators conform to different regulations and standards. Take the N95 masks as an example. They are disposable products under class II and approved by both the FDA and NIOSH. N95 masks provide nearly 100% protection from particles ranging from large droplets (>100,000 nm) to inhalable droplets (10−100,000 nm) and nuclear aerosols (<10,000 nm) [47,48,49], and they can filter out at least 95% of particles as small as 300 nm in the air. Moreover, N95 masks can effectively filter airborne biological particles, such as viruses and bacteria, making them irreplaceable in the fight against viruses transmitted through the respiratory tract, such as COVID-19 and SARS-CoV-2.

### 3.4. Functional Face Masks

In addition to the three main types of masks mentioned above, various masks for the general public are currently available or under development to achieve novel functions.

#### 3.4.1. Antibacterial

Antibacterial masks have been widely developed to inhibit the survival of microorganisms and protect the wearers. Several well-known antibacterial agents have been utilized for infection control, such as metal nanoparticles [50] and metal oxide [37]. They are non-toxic, compatible with skin microbiota, and effectively inhibit against a broad spectrum of bacteria, which makes them suitable for applying to masks [45]. It has been revealed by Wang et al. [51] that Ag nanoparticles are effective in killing viruses and bacteria. They fabricated Ag/PI membrane by adding Ag nanoparticles to the original fiber membrane and found that the newly prepared Ag/PI membrane could effectively remove *Escherichia coli* and *Staphylococcus*. Xing et al. [52] inoculated the expanded polytetrafluoroethylene (ePTFE) fibrous filters with ZnO nanorods. The ZnO was able to curb the reproduction of Gram-negative and positive bacteria.

#### 3.4.2. Biodegradability

As conventional masks are single-used and non-biodegradable, a large number of discarded masks have posed massive polymeric wastes. Therefore, masks are expected to be made of biodegradable materials to abate plastic pollution. Various bio-based filtration media, made of chitosan, polylactic acid, and cellulose, have been patented and launched as biodegradable surgical masks in recent years [45].

#### 3.4.3. Others

Under severe pandemic conditions, more functions make for a better PPE package [53]. As reported, several fabrications of novel electret filter structures, combined with bulky and open systems, allow for recharging and rejuvenating after disinfection, which is conducive to the development of reusable masks. Furthermore, the thermal comfort filter layers, consisting of polysulfonamide/polyacrylonitrile−boehmite (PSA/PAN−B) composites [45,54], are highly breathable. Yang et al. [55] reported a new barium titanate @polyurethane/polysulfonamide (BaTiO_3_@PU/PSA) composite nanofibrous membranes that have shown favorable flame-retardancy and sufficient flexibility. Ding et al. [56] have developed highly transparent nanofibrous membranes used as transparent masks.

## 4. Nanofibers

**Figure 2:**
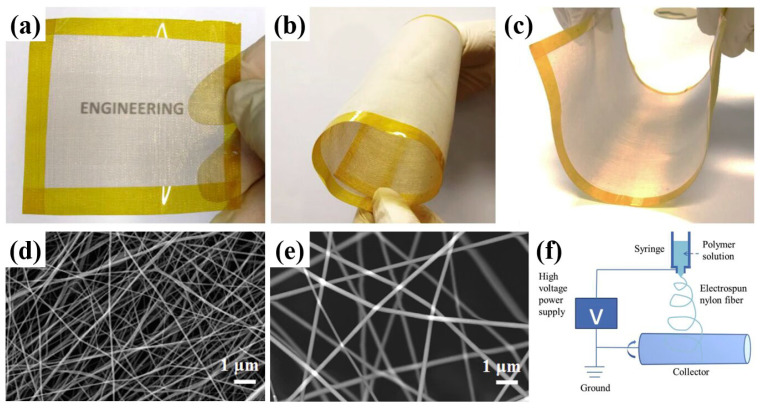
(**a**–**c**) Photographs of a fabricated nanofiber filter that was thin, transparent, and flexible [71]. (**d**) SEM image of the nanofiber filters with an electrospinning time of 3 h and a nylon concentration of 18%. (**e**) SEM image of PVA electrospun nanofibers [72]. (**f**) Schematic of the experimental setup for the electrospinning process [71].

Nanofiber (in Figure 2a–c) refers to the fiber with a diameter in nanoscale and a large length with a certain aspect ratio. Generally, the diameter of nanofibers is between 1 nm and 100 nm, as shown in Figure 2d,e; however, fibers with a diameter below 1000 nm can also be defined as nanofibers [57]. Nanofibers possess several unique properties and display a quantity of advantages over microfibers, such as [58,59] being lightweight and having high strength, high stiffness, and a large surface area.

Nanofibers exhibit special properties, mainly due to extremely high surface-to-weight ratio and extremely small size [60]. Additionally, it is possible to modify the nanofibers with external physi-chemical properties [61], which make them appropriate for a wide range of applications [62,63], such as environmental, biomedical, and electronic applications.

The electrospinning technique is a facile spinning method for preparing nanofibers by using electrostatic field force [64]. Among various nanofiber fabrication methods, electrospinning has been considered the advanced and extensively used technique [65,66]. An electrospinning unit is composed of a syringe pump, a spinneret, a high voltage supplier, and a collector [67] (in Figure 2f). The morphology and structure of nanofibers can be regulated by polymer solutions, operating parameters [68], and environmental parameters, including temperature and humidity [69].

Due to the versatility of the electrospinning technique, a large number of candidates can be selected for the fabrication of electrospun nanofibers, which include synthetic and natural polymers, metals and oxides, carbon-based materials, etc. [70].

## 5. Biodegradable Nanofiber Filters

Biodegradable materials can be classified into natural and synthetic materials [73] (e.g., polyvinyl alcohol (PVA), polyethylene oxide (PEO), and polyvinyl pyrrolidone (PVP), etc.). Synthetic materials have better mechanical properties, while natural ones are more biocompatible, which makes them have much more potential to be filtering materials for making facemasks.

### 5.1. Silk Fibroin

Silk, which is produced by silkworms, has been commonly used in the textile application for thousands of years, due to its several excellent properties, such as its lightweight features, comfort, flexibility, etc. [74]. There are two types of proteins in silk, which include silk fibroin and silk sericin, respectively. Generally, the percentage of silk fibroin is almost 70–80%, and the rest of silk sericin accounts for 20–30%. As the major component of silk, SF consists of three major proteins: heavy chains (H chains), light chains (L chains), and glycoprotein of P25. The molecular weight of heavy and light chains is about 391 and 26 kDa, respectively. Heavy and light chains are linked with disulfide bonds to form the H-L complex. While the glycoprotein of P25 is hydrophobically linked to H-L complex [75,76]. Generally, sericin is removed by the treatment of a degumming process under boiling alkaline conditions, resulting in only silk fibroin being left [77]. SF exhibited outstanding properties, such as excellent biocompatibility and biodegradability, low toxicity, low cost, and mechanically robust strength, which ensures it as a promising candidate to be used in various applications [78,79,80,81]. Since the high specific surface area and network structure of nanofiber membrane can control PM effectively, it is meaningful to develop a SF nanofiber filter for effective PM control.

**Figure 3:**
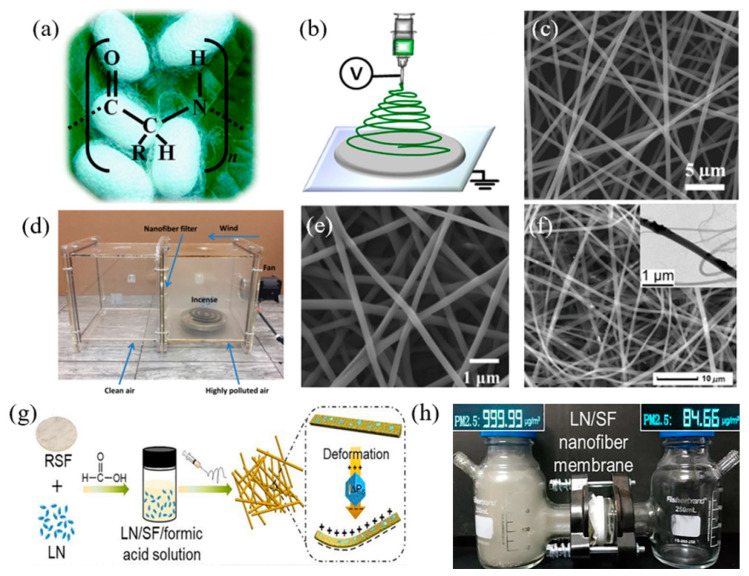
(**a**) Molecular formula of silk protein made from silkworm cocoons. (**b**) Schematic diagram of the preparation of SF nanofibers by electrostatic spinning. (**c**) SEM image of SF nanofibers [82]. (**d**) Simulated filtration demonstration of SF/PVA composite electrostatic spun nanofiber filter set blocking PM contamination diffusion [83]. (**e**) SEM image of SF/PVA nanofiber filters with the diameter of around 240 nm. (**f**) SEM image of LN/SF nanofiber membranes. The inserted image shows the transmission electron microscopy image of LN/SF nanofiber. (**g**) Schematic diagram of the process of preparing LN/SF nanofiber films. (**h**) Demonstration of air filtration with commercial haze detectors simulating LN/SF nanofiber membranes [81].

As a natural protein, SF contains plenty of functional groups (in Figure 3a), which can enhance the interaction between SF nanofibers and tiny particles. Wang et al. [82] first developed a lightweight SF nanofiber filter with an electrospinning method for air filtration (in Figure 3b,c). The free-standing SF nanofiber air filter exhibited a high PM_2.5_ filtration efficiency of 98.8%. Furthermore, Bian et al. [83] blended SF into degradable PVA solutions to develop composite electrospun SF/PVA nanofiber filters for enhanced PM capture (in Figure 3d). As can be seen in Figure 3e, the diameter of SF/PVA is around 240 nm. The filtration results confirmed that the developed SF/PVA air filter achieved an extremely high filtration efficiency of 99.11%, which was 10% higher than the pure PVA nanofiber filter. In addition, Alena et al. used the electrospinning method to develop recycled poly (ethylene terephthalate)/silk fibroin (r-PSF) composite fibrous membranes for air filtration [84]. The air permeability of the composite filter can be improved with the increased amount of SF. The developed filter also captured PM effectively, as the filter with the basis weight of 11.15 g·m^−2^ exhibited a filtration efficiency of 99% and a quality factor of 0.024 Pa^−1^. Though SF or SF-based nanofiber filters can achieve superior filtration performance, it is still a challenge to improve the mechanical performance. To enhance the mechanical property of the SF membrane, Wang et al. [81] developed a flexible electrospun SF membrane doped with piezoelectric LiNbO_3_ nanoparticles. The preparation process is shown in Figure 3g, and the scanning electron microscope (SEM) of prepared LN/SF nanofiber membranes is in Figure 3f. Moreover, the adoption of LiNbO_3_ nanoparticles can enhance the filtration performance of the composite filter based on the electrostatic interaction between the filter and PM pollutants. Figure 3h visualizes the filtration performance of the LN/SF nanofiber membranes.

SF fibers represent outstanding mechanical properties. The aforementioned heavy chains can form stable anti-parallel β-sheet structures, giving SF rigidity and strength, while light chains form amorphous substrates with a low degree of order [85]. The rich secondary structure leads to stability and ultimate performance of SF, which not only provides excellent mechanical strength, but also gives stability in the water.

### 5.2. PLA

Polylactic acid (PLA) is a biodegradable material derived from renewable plant resources, such as corn and sugar cane [86,87]. Owing to its excellent biodegradability and biocompatibility, PLA has been mostly used in the biomedical field. Recently, PLA has also been regarded as ideal filtration media for achieving outstanding filtration performance.

**Figure 4:**
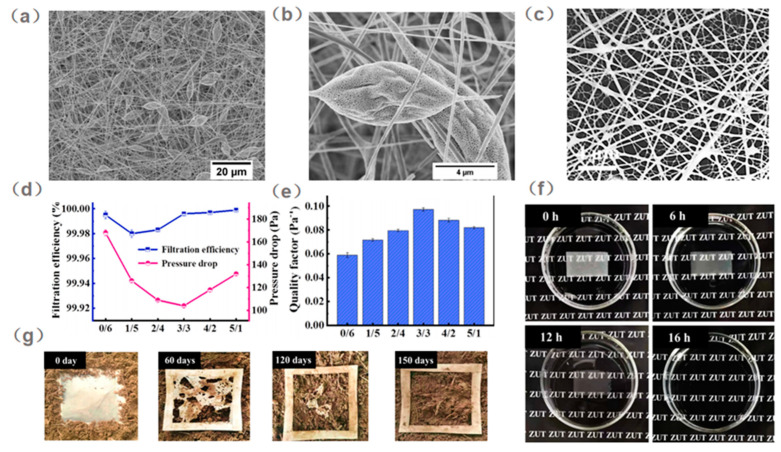
SEM images of (**a**) the porous bead-on-string PLA fibers fabricated from 5 wt% PLA solutions in 10/1 DCM/DMAC by weight, and (**b**) a corresponding highly magnified image, and (**c**) multi-scale structured membranes with the number of spinning unit ratio of 2/4 [92]. (**d**) Filtration performance and (**e**) quality factor of multi-scale structured membranes with the different number of spinning unit ratios of TN−PLA to S−PLA. (**f**) Time-dependent enzymatic degradation images of the MSM−PLA (3/3, 15 mg) and (**g**) Images showing the soil burial degradation of the MSM−PLA (3/3, 40 mg) under natural conditions [93].

During electrospinning, porous beaded fibers can be constructed. The developed membrane has a large specific surface area, making high air filtration efficiency possible [88,89]. In addition, nanopores on the surface of the beads help to adsorb the particles [90,91]. Inspired by this fiber morphology, Wang et al. [92] developed porous bead-on-string PLA nanofiber filters for PM control. The bead-like fiber can be controlled by the regulation of the solvent compositions and PLA concentration. The results showed that the larger bead size and higher BMR value reduced the volume fraction of the membrane, which reduced the pressure drop. In addition, the fiber with a small diameter and the additional mesopores in the beads could facilitate the capture of particles. The optimized nanofibrous filter with PLA of 5 wt% can achieve an extremely high filtration efficiency of 99.997% and a relatively low pressure drop of 165 Pa, as shown in Figure 4a–c. Furthermore, Wang et al. [93] designed a multiscale structured electrospun PLA nanofibrous membrane. The resultant filter was composed of two layers, one is the true nanoscale PLA nanofiber with a diameter of 37 nm, and the other is the support layer of PLA nanofiber with a diameter of 187 nm. This novel structure enables the PLA filter to exhibit superior filtration performance, achieving a high PM_0.3_ removal efficiency of 99.996% and a low pressure drop of 104 Pa, as shown in Figure 4d,e.

PLA electrospun air filters also showed excellent biodegradability, which is superior to single-use face masks. Wang et al. [93] confirmed the enzymatic and natural degradation of PLA nanofibers. As shown in Figure 4f,g, the filter was completely decomposed with enzymatic degradation after 16 h. When the PLA air filter was buried in the soil, it would take 150 days for the final degradation. Compared with commercial masks or respirators, the use of PLA as personal protective equipment is expected to alleviate the environmental pollution and resource waste caused by abandoned masks, paving the way for the protection of the ecosystem.

Overall, mask filters using PLA as a raw material exhibited comprehensive properties, including robust PM removal capability and low air resistance. More importantly, PLA nanofiber filters have been confirmed to show great biodegradability, which could serve as green candidates for the development of protective equipment.

### 5.3. Chitosan

**Figure 5:**
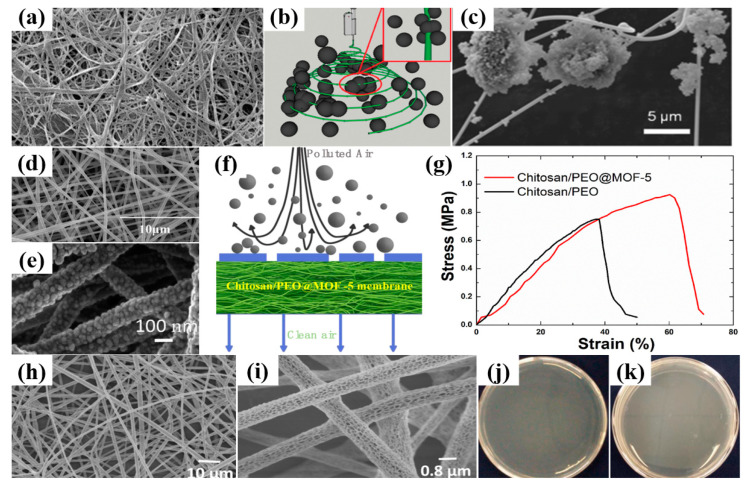
(**a**) SEM micrograph of chitosan nanofibrous matrix [104]. (**b**) Schematic illustration of the mechanism for high-efficiency PM_2.5_ capture using polymer nanofibers via in situ electrospinning. Inset: deposition of PM_2.5_ on the surface of the nanofibers with high magnification. (**c**) SEM image of the electrospun chitosan nanofibers in the polluted air [96]. (**d**) SEM photograph of HTCC nanofiber membrane [98]. (**e**) High magnification SEM of chitosan/PEO@MOF-5 membrane, growth of time 30 s. (**f**) Schematic representation of the polluted air passing through chitosan/PEO@MOF-5 filter for PM removal; (**g**) The stress–strain curves of the freestanding electrospun chitosan/PEO membrane and chitosan/PEO@MOF-5 membrane [99]. (**h**,**i**) FE-SEM images of the PLA/chitosan fibrous membranes prepared at concentration: 8 wt% PLA, 2 wt% CS. (**j**) Antibacterial activity of the fibrous membranes against S. aureus. The samples fibrous are membranes with 8 wt% PLA and 2.5 wt% CS. (**k**) Antibacterial activity of the fibrous membranes against E. coli. The samples are fibrous membranes with 8 wt% PLA and 2.5 wt% CS [100].

Chitosan is a polysaccharide acquired by the N-deacetylation of chitin, and it is made of glucosamine and N-acetylglucosamine units [94]. Owing to its biological non-toxicity, excellent biodegradability, biocompatibility, and antibacterial properties, it has considered a green natural macromolecule material for environmental protection [95]. Additionally, the advantages of chitosan make it a research hotspot in biomedical applications [73]. Due to the versatility of electrospinning, it is also desirable to develop chitosan nanofibers, as shown in Figure 5a. Recently, chitosan has been selected as a promising filtration material for enhancing PM removal. For example, Zhang et al. [96] proposed a facile method of in situ electrospinning of chitosan nanofibers directly into a polluted environment. Owing to the strong polarity and positively charged functional groups on chitosan, the developed chitosan nanofiber can capture PM effectively (in Figure 5b,c). Moreover, the lightweight property of chitosan electrospun fiber could provide more opportunities to combine with PM.

Owing to the low stability and high viscosity of chitosan solutions, it is also common to employ blending polymers to improve the processability of electrospun nanofibers. The compatibly used blending polymers could be polyurethane (PU), polyvinyl alcohol (PVA), and polyethylene (PEO), etc. [97]. For example, Wang et al. [98] used PVA to blend with quaternized chitosan for the development of composite electrospun nanofiber (in Figure 5d). The proposed chitosan/PVA air filter exhibited a filtration efficiency of 86% for PM_2.5_. To enhance the filtration performance, Pan et al. [99] used an electrospinning technique to produce chitosan/PEO nanofiber first (in Figure 5e). Then, they embedded the growth of MOF-5 on the platform of composite chitosan/PEO membranes. The functionalized chitosan-based air filter exhibited a high PM_2.5_ removal efficiency of 99.95% and an initial pressure drop of 44 Pa (in Figure 5f). The addition of MOF-5 not only enhances the overall filtration performance, but also improves the mechanical property, compared with the pure chitosan/PEO filter. For instance, the Young modulus of the chitosan/PEO@MOF-5 membrane is 2.53 MPa, which is much larger than that of chitosan/PEO membrane (in Figure 5g). Therefore, it can be seen that a load of functional materials in a chitosan-based membrane can improve the overall filtering performance.

Moreover, Li et al. [100] used a one-step electrospinning technique to fabricate PLA/chitosan composite air filters with highly porous structures, as shown in Figure 5h,i. The proposed environmentally friendly composite fibrous filter achieved a high removal efficiency of 98.99% for particles around 300 nm and a relatively low pressure drop of 148 Pa. The developed filter also demonstrated excellent antibacterial performance for *Escherichia coli* at 99.4% and *Staphylococcus aureus* at 99.5%, respectively (in Figure 5j,k).

Chitosan provides many advantages and disadvantages. As a biomaterial form, chitosan can be made into the forms of hydrogels, sponges, and films, which appear in 3D forms, as well as a porous membrane, which appears in the 2D form and exhibits specific applications [101,102]. Generally, chitosan nanofibers can be developed into filters with high porosity and tunable pore structure, which is applicable to be used for air filtration [103].

### 5.4. Cellulose

Cellulose, which is derived from plants and marine animals [105], is a macromolecular polysaccharide composed of glucose [106]. Its fibrous structure plays an essential role in the structure of plant cell walls [107]. As a carbohydrate polymer, the molecular structure enables the cellulose specific properties. Due to its versatility and biodegradability, cellulose can be regarded as a potential material for the development of disposable membranes. The size of the nanocellulose and its high specific surface area (up to 101.8 m^2^/g) [108] provide thin filter media with high porosity and better breathability, as well as an ability to capture and absorb small particles. In addition, with the aid of physical or chemical modification, cellulose fiber also achieves various functional groups.

**Figure 6:**
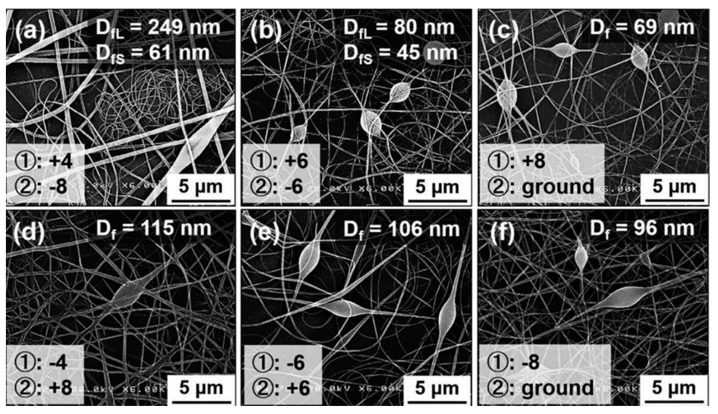
SEM images of the spun precursor containing 8 wt% PVP and 0.2 wt% CNFs with various needle and collector voltages: (**a**) 4 and−8 kV, (**b**) 6 and−6 kV, (**c**) 8 kV and ground, (**d**) −4 and 8 kV, (**e**) −6 and 6 kV, and (**f**) −8 kV and ground [110].

Ma et al. [109] studied the feasibility of using an electrospinning technique to prepare cellulose nanofiber net from cellulose acetate (CA) solution. The diameter of the developed CA nanofiber ranged from 200 nm to 1 μm. Hierarchically structured nanofibers are gaining attention in the field of the filter. Balgis et al. [110] accomplished the formation of well-mixed nano- and submicron-size cellulose−polyvinylpyrrolidone (PVP) nanofiber composites. Figure 6 shows that the Taylor cone stability was affected by the sign and value of the voltage applied to the needle. The application of a positive voltage to the needle may affect the stability of the Taylor cone. Aerosol filtration measurements show that the multilayer air filter has an incredibly high performance, as shown by the high QF of 0.117 Pa^−1^, which is 10 times that of commercial HEPA filters.

**Figure 7:**
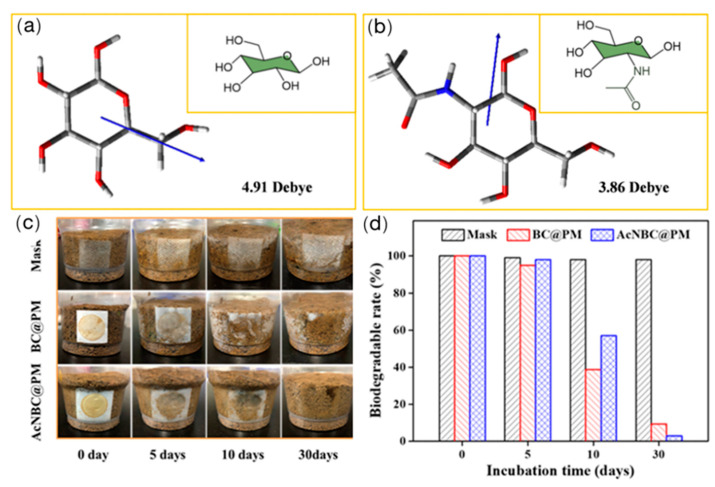
(**a**,**b**) Molecular model and the corresponding dipole moments of BC and AcNBC. (**c**) Photographs of the commercial mask, BC, and AcNBC filters at different incubation times in the soil. (**d**) Biodegradable rate of the BC, AcNBC filters, and commercial masks by calculating the area of the filters [111].

Sun et al. [111] developed an in situ approach to modify bacterial cellulose (BC) into N-acetyl BC (AcNBC) as filters for the removal of PM (in Figure 7a,b). The filtration results indicated that an AcNBC filter showed a higher PM removal efficiency than that of pristine BC and met the qualification of the high-efficiency standard of 95% under an environment with a high concentration of PM. Moreover, the BC-based filters after filtration showed favorable biodegradation in a real soil environment, as shown in Figure 7c,d. The degradation of the cellulose-based membrane was also confirmed by Hardian et al. [112]. The developed cellulose/chitosan membrane showed good degradability under enzyme degradation.

In general, cellulose contains hydrogen bonds and is insoluble in neither water nor ordinary organic solvents, so it is relatively stable at room temperature. Cellulose molecules are polar and interact strongly with each other, belonging to the semi-rigid material. The tensile strength, flexural strength, and dimensional stability of cellulose are stronger with a greater degree of crystallinity. Additionally, cellulose has excellent biodegradability and a high specific surface area, which can support it as a good filtration material.

### 5.5. Zein

Zein is the main storage protein of maize, commonly extracted from corn endosperm [113]. As a plant-derived protein, it has been widely utilized in the food and biomedical industry because of its biocompatibility and biodegradability [114]. Additionally, zein possesses abundant functional groups [115], which can facilitate the interaction with air pollutants (such as PM) [116,117]. Moreover, the excellent film-forming ability gives zein broad application prospects in the field of air filtration.

**Figure 8:**
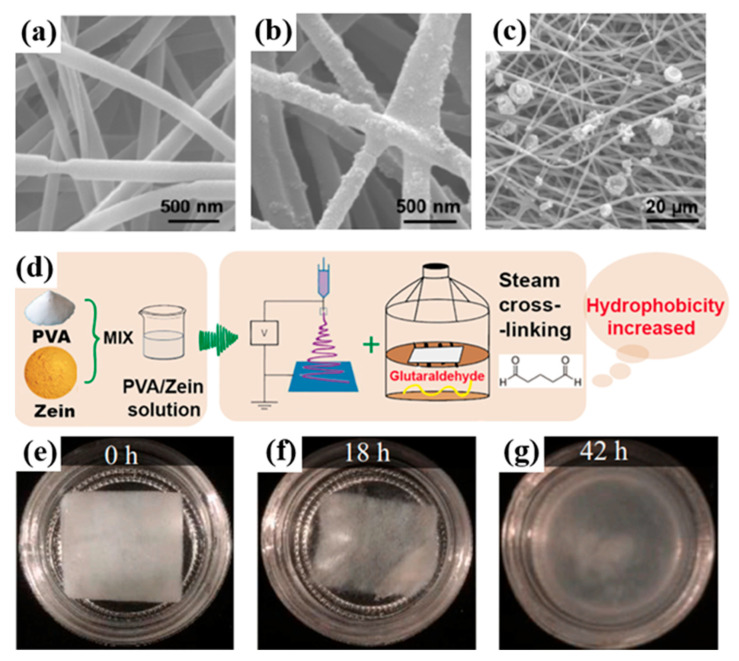
(**a**) SEM image of zein nanofiber membrane before filtration. (**b**,**c**) SEM images of zein nanofiber membrane after filtration [118]. (**d**) Schematic preparation and design of the filter based on hydrophobic cross-linked zein nanofibers [119]. (**e**–**g**) Images of enzymatic degradation testing [120].

In recent years, the development of zein electrospun nanofibers as air filtration media has been widely studied. For example, Tian et al. [118] demonstrated zein nanofiber membranes as high-performance filters for PM control. The zein air filter was fabricated with the aid of polyethylene oxide (PEO), which could be blended with zein for the development of electrospun nanofibers (in Figure 8a–c). The hydrophobicity of zein embeds the filter’s excellent water-resistance. Regarding the filtration performance, the fabricated zein air filter showed a high removal efficiency of 99.5% for submicron particles. To further improve the moisture resistance of the zein filter, Tian’s group [119] used glutaraldehyde to cross-link with zein-based nanofibers (in Figure 8d). The cross-linked zein membrane also achieved improved mechanical properties.

**Figure 9:**
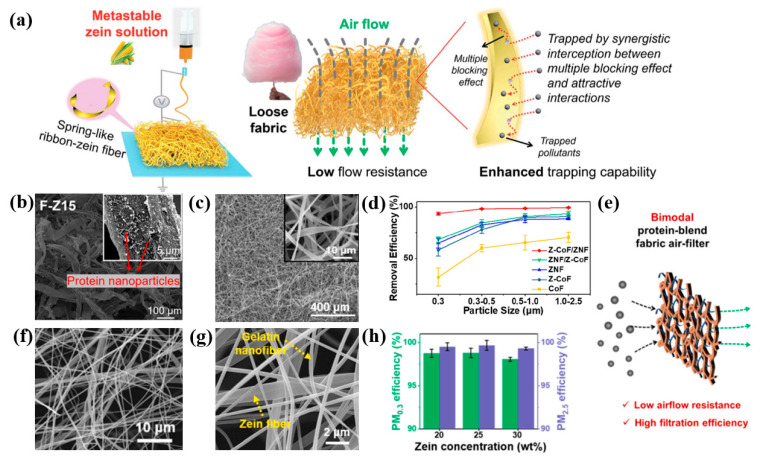
(**a**) Schematic showing metastable zein solution generating cotton-candy-like loose fabric [121]. (**b**) SEM image of F-Z15 air filter [116]. (**c**) SEM image showing the morphology of zein nanofiber. (**d**) Removal efficiencies for particles at different sizes of various air filters [122]. (**e**) Schematic of a bimodal protein-blend fabric. (**f**,**g**) SEM images of bimodal protein-blend fabric. (**h**) PM_2.5_ and PM_0.3_ removal efficiencies of the protein-blend fabric air-filters prepared with different concentrations [123].

For the design of an efficient zein-based air filter, it is appealing to modify the fiber morphology and membrane structure. For example, Hu et al. [120] steered the fiber morphology by controlling the ratio of mixture solvent and obtained the round or flat ribbon zein fibers. They also conducted a biodegradation test and confirmed that the zein fiber was almost decomposed within 42 h via enzymatic degradation, as shown in Figure 8e–g. Additionally, Fan et al. [121] developed a metastable zein solution to fabricate nanofibers with ribbon morphology (Figure 9a). The proposed cotton-candy-like zein filter with a loose structure achieved superior filtration performance in both pressure drop and filtration efficiency. Furthermore, they [116] fabricated a hierarchically structured filter by exploiting wood pulp (WP) as a frame to hold cellulose nanofibers (CNF)/zein nanoparticles. The developed composite filter exhibited a high-performance for PM control, as WP fibers reduced the pressure drop and the functional CNF/zein nanoparticles improved the filtration efficiency (in Figure 9b). In addition, Liu et al. [122] fabricated a bi-layered air filter, shown in Figure 9c, combining a thin layer of protein nanofibers with the zein-functionalized cotton fibers via an evaporation-controlled strategy, which showed a remarkable PM filtration efficiency of 99%. Fu et al. [123] designed a bimodal protein-blend filter, as shown in Figure 9e via multi-jet electrospinning. The developed gelatin/zein composite air filter (in Figure 9f,g) exhibited remarkable filtration efficiencies of 99.67% for PM_2.5_ and 98.80% for PM_0.3_ (in Figure 9h), yielding an ultra-low pressure drop of 38 Pa.

Due to the abundant functional groups and superior film-forming ability, zein-based nanofibers have a good potential as “green” materials for multifunctional and high-efficiency air filtration materials [119]. Additionally, zein is gaining more attention because of its multifunctionality and biodegradability. However, an optimal solvent system and a much greener and simpler electrospinning process are still under development.

## 6. Conclusions

The ongoing respiratory disease has posed a great threat to human health, which sparked a huge consumption of personal protective equipment (e.g., face masks). In this review, we have emphasized the significance of the development of biodegradable nanofiber filters for efficient PM capture. The filtration mechanisms of fibrous filters, including inertia, diffusion, interception, gravity, and electrostatic interaction, are introduced in detail. Regarding the evaluation of filtration performance, several parameters, including filtration efficiency, pressure drop, and quality factor, would be used for the assessment. Furthermore, the current personal protective equipment of masks is discussed. The discarded masks can generate a large amount of plastic waste to endanger the ecosystem, which makes it urgent for the development of biodegradable membranes as face masks. Here, we have summarized the most attractive biodegradable nanofiber filters and discussed their fabrication, characterization, and filtration performance (in Table 1).

Though biodegradable nanofiber filters have been considered promising candidates for PM capture, there still existed several challenges that need to be addressed. The currently used biodegradable filters are limited, so it is necessary to discover more types of biodegradable materials for effective PM capture. Instead of using a single biodegradable component to fabricate nanofiber membranes, an air filter with multi-components is the development trend in the future. The resulting composite filter could exhibit outstanding filtration performance, to which it can be ascribed that the doped functional materials embedding the filter achieve the optimized filter structure and more physic-chemical properties. Additionally, the degradable filters tend to be degraded, and the long-term stability should be taken into consideration. Furthermore, the fabrication process of biodegradable filters still needs simplification

Nanofiber filtration membranes made of biodegradable materials have many advantages, but there are also defects that need attention. Currently, the most severe obstacle to biodegradable materials is their production costs. Because of the complex extraction process from nature, the production costs of biodegradable materials may be more expensive than those of similar conventional plastics or recycled plastics. Secondly, many biodegradable membranes only degrade quickly under certain conditions. For example, PLA is one of the most productive degradable materials and can only achieve rapid degradability under enzymatic conditions.

Owing to growing awareness of the negative effects of traditional practices on the environment and health, green manufacturing has emerged across industries. Electrospinning is a ubiquitous manufacturing method in the biomaterials industry for producing nanoscale to microscale fiber webs that resemble natural tissues, but the process has traditionally used solvents that are environmentally harmful, and poses a significant barrier to industrial expansion and clinical conversion. Therefore, as a new production technology, green electrospinning has relatively broad development and application prospects. Mosher et al. [124] developed the green electrospinning process with acetic acid as the green solvent by systematic testing of bio-benign solvents. The resulting green fibers and composites are comparable to conventional grids, in terms of composition, chemistry, structure, mechanical properties, and biocompatibility. Interestingly, the material properties of green synthetic fibers are more in imitation than traditional electrospun fibers, doubling their ductility. David et al. [125] prepared collagen/hydroxyapatite composite nanofiber scaffolds by gentle solvent electrospinning. They had shown that green electrospinning could be used to generate nanocomposite scaffolds with potential biomedical applications. Furthermore, green electrospinning also focuses on the perspective of materials. The materials reviewed in this paper are green and pollution-free and non-chemically synthesized, such as silk fibroin (SF), polylactic acid (PLA), chitosan, cellulose, and zein. In addition, the biodegradable nature of these materials is also green and pollution-free.

Furthermore, biodegradable electrospun nanofibers have the potential to be developed into smart face masks. Smart material needs innovative technology. Fibers with a high surface-to-volume ratio, high porosity, exceptional mechanical properties, and stability can be fabricated by the electrospinning technique [126], which is versatile and applied to a wide range of raw materials, making it possible to design functional devices with unique chemical–physical characteristics. Sio et al. [127] reported how to realize non-disposable and highly comfortable respirators with a light-triggered self-disinfection ability by bridging bioactive nanofiber properties and stimuli-responsive nanomaterials. Additionally, the feasibility of developing smart face masks with biodegradable materials has been initially verified [128]. Overall, degradable nanofiber filters would be of great significance for the design of green face masks.

## Figures and Tables

**Table 1:** Summary of biodegradable nanofiber filters for air filtration.

Materials	PM/μm	Air Velocity	FE/%	Pressure Drop/Pa	QF/Pa^−1^	Ref
LiNbO_3_/SF	0.3–10	0.142 m/s	98.1	102	0.039	[81]
SF	2.5	0.531 m/s	98.8	98	0.045	[82]
SF/PVA	2.5	0.5 m/s	99.11	50	0.094	[83]
r-PET/SF	0.12–2.46	0.053 m/s	92.93	61.2	0.043	[84]
PLA	0.260	0.058 m/s	99.997	165.3	0.064	[92]
PLA	2.5	32 L/min	99.996	104	0.097	[93]
Chitosan/PVA	2.5	0.5 m/s	86	52	0.038	[98]
Chitosan/PEO	2.5		99.95	44	0.0737	[99]
Chitosan/PLA	0.3	0.014 m/s	98.99	147.60	0.031	[100]
Cellulose/PVP	0.3–0.5		86.4	17	0.117	[110]
Cellulose	0.3–10	0.02 m/s	95	120	0.025	[111]
Zein	0.3–10		99.6	175	0.032	[118]
Zein	0.3	0.0533 m/s	99	109	0.042	[120]
Zein/gelatin	2.5	0.062 m/s	99.67	38	0.150	[123]

## Data Availability

Not applicable.

## References

[1] Yan E., Lai D.W.L., Lee V.W.P., Ng H.K.L. (2021). Predicting Public Adherence to COVID-19 Preventive Measures: A Cross-Sectional Study in Hong Kong. Int. J. Environ. Res. Public Health.

[2] Cherrie J.W., Apsley A., Cowie H., Steinle S., Mueller W., Lin C., Horwell C.J., Sleeuwenhoek A., Loh M. (2018). Effectiveness of face masks used to protect Beijing residents against particulate air pollution. Occup. Environ. Med..

[3] Roberge R.J., Kim J.-H., Coca A. (2012). Protective Facemask Impact on Human Thermoregulation: An Overview. Ann. Occup. Hyg..

[4] Wang C.C., Prather K.A., Sznitman J., Jimenez J.L., Lakdawala S.S., Tufekci Z., Marr L.C. (2021). Airborne transmission of respiratory viruses. Science.

[5] Benson N.U., Bassey D.E., Palanisami T. (2021). COVID pollution: Impact of COVID-19 pandemic on global plastic waste footprint. Heliyon.

[6] Dissanayake J., Torres-Quiroz C., Mahato J., Park J. (2021). Facemasks: A Looming Microplastic Crisis. Int. J. Environ. Res. Public Health.

[7] Bian Y., Niu Z., Wang S., Pan Y., Zhang L., Chen C. (2022). Removal of Size-Dependent Submicron Particles Using Metal-Organic Framework-Based Nanofiber Air Filters. Acs Appl. Mater. Interfaces.

[8] Niu Z., Bian Y., Xia T., Zhang L., Chen C. (2021). An optimization approach for fabricating electrospun nanofiber air filters with minimized pressure drop for indoor PM2.5 control. Build. Environ..

[9] Wang L., Bian Y., Lim C.K., Niu Z., Lee P.K.H., Chen C., Zhang L., Daoud W.A., Zi Y. (2021). Tribo-charge enhanced hybrid air filter masks for efficient particulate matter capture with greatly extended service life. Nano Energy.

[10] Bian Y., Wang R., Wang S., Yao C., Ren W., Chen C., Zhang L. (2018). Metal-organic framework-based nanofiber filters for effective indoor air quality control. J. Mater. Chem. A.

[11] Bian Y., Chen C., Wang R., Wang S., Pan Y., Zhao B., Chen C., Zhang L. (2020). Effective removal of particles down to 15 nm using scalable metal-organic framework-based nanofiber filters. Appl. Mater. Today.

[12] Deng Y., Lu T., Cui J., Samal S.K., Xiong R., Huang C. (2021). Bio-based electrospun nanofiber as building blocks for a novel eco-friendly air filtration membrane: A review. Sep. Purif. Technol..

[13] He P.W., Wu F., Yang M., Jiao W.L., Yin X., Si Y., Yu J.Y., Ding B. (2021). Green and antimicrobial 5-bromosalicylic acid/polyvinyl butyral nanofibrous membranes enable interception-sterilization-integrated bioprotection. Compos. Commun..

[14] Bian Y., Zhang C., Wang H., Cao Q. (2023). Degradable nanofiber for eco-friendly air filtration: Progress and perspectives. Sep. Purif. Technol..

[15] Jalvandi J., White M., Gao Y., Truong Y.B., Padhye R., Kyratzis I.L. (2017). Polyvinyl alcohol composite nanofibres containing conjugated levofloxacin-chitosan for controlled drug release. Mater. Sci. Eng. C-Mater. Biol. Appl..

[16] Kadam V., Kyratzis I.L., Truong Y.B., Wang L.J., Padhye R. (2020). Air filter media functionalized with beta-Cyclodextrin for efficient adsorption of volatile organic compounds. J. Appl. Polym. Sci..

[17] Truong Y.B., Glattauer V., Briggs K.L., Zappe S., Ramshaw J.A.M. (2012). Collagen-based layer-by-layer coating on electrospun polymer scaffolds. Biomaterials.

[18] Sinha-Ray S., Zhang Y., Yarin A.L., Davis S.C., Pourdeyhimi B. (2011). Solution Blowing of Soy Protein Fibers. Biomacromolecules.

[19] Song W., Zhang M., Huang X., Chen B., Ding Y., Zhang Y., Yu D.G., Kim I. (2022). Smart l-borneol-loaded hierarchical hollow polymer nanospheres with antipollution and antibacterial capabilities. Mater. Today Chem..

[20] Khansari S., Duzyer S., Sinha-Ray S., Hockenberger A.S., Yarin A.L., Pourdeyhimi B. (2013). Two-Stage Desorption-Controlled Release of Fluorescent Dye and Vitamin from Solution-Blown and Electrospun Nanofiber Mats Containing Porogens. Mol. Pharm..

[21] Tang Y.X., Varyambath A., Ding Y.C., Chen B.L., Huang X.Y., Zhang Y., Yu D.G., Kim I., Song W.L. (2022). Porous organic polymers for drug delivery: Hierarchical pore structures, variable morphologies, and biological properties. Biomater. Sci..

[22] Sett S., Lee M.W., Weith M., Pourdeyhimi B., Yarin A.L. (2015). Biodegradable and biocompatible soy protein/polymer/adhesive sticky nano-textured interfacial membranes for prevention of esca fungi invasion into pruning cuts and wounds of vines. J. Mater. Chem. B.

[23] Tien C. (2012). Principles of Filtration.

[24] Pellegrino J. (2000). Filtration and ultrafiltration equipment and techniques. Sep. Purif. Methods.

[25] Gao Y., Tian E., Zhang Y., Mo J. (2022). Utilizing electrostatic effect in fibrous filters for efficient airborne particles removal: Principles, fabrication, and material properties. Appl. Mater. Today.

[26] Tian E., Yu Q., Gao Y., Wang H., Wang C., Zhang Y., Li B., Zhu M., Mo J., Xu G. (2021). Ultralow Resistance Two-Stage Electrostatically Assisted Air Filtration by Polydopamine Coated PET Coarse Filter. Small.

[27] Wang Z.H., Zhu X.W., Cheng X.X., Bai L.M., Luo X.S., Xu D.L., Ding J.W., Wang J.L., Li G.B., Shao P.H. (2021). Nanofiltration Membranes with Octopus Arm-Sucker Surface Morphology: Filtration Performance and Mechanism Investigation. Environ. Sci. Technol..

[28] Sabit A. (2017). Wellington Sears Handbook of Industrial Textiles.

[29] Maduna L., Patnaik A. (2017). Textiles in air filtration. Text. Prog..

[30] Bandi M.M. (2020). Electrocharged facepiece respirator fabrics using common materials. Proc. R. Soc. A-Math. Phys. Eng. Sci..

[31] Stechkina I.B., Kirsch A.A., Fuchs N.A. (1969). Studies on fibrous aerosol filters. IV. Calculation of aerosol deposition in model filters in the range of maximum penetration. Ann. Occup. Hyg..

[32] Lee K.W., Liu B.Y.H. (1982). Theoretical-Study of Aerosol Filtration by Fibrous Filters. Aerosol Sci. Technol..

[33] Gao Y., Tian E., Mo J. (2023). Electrostatic Polydopamine-Interface-Mediated (e-PIM) filters with tuned surface topography and electrical properties for efficient particle capture and ozone removal. J. Hazard. Mater..

[34] Chen R., Zhang K., Wang H., Wang X.M., Zhang X.H., Huang X. (2022). Incorporating catalytic ceramic membrane into the integrated process of in situ ozonation, membrane filtration and biological degradation: Enhanced performance and underlying mechanisms. J. Membr. Sci..

[35] Sanyal A., Sinha-Ray S. (2021). Ultrafine PVDF Nanofibers for Filtration of Air-Borne Particulate Matters: A Comprehensive Review. Polymers.

[36] Stanislas T.T., Bilba K., Santos R.P.d.O., Onesippe-Potiron C., Savastano Junior H., Arsene M.-A. (2022). Nanocellulose-based membrane as a potential material for high performance biodegradable aerosol respirators for SARS-CoV-2 prevention: A review. Cellulose.

[37] Liu H., Cao C., Huang J., Chen Z., Chen G., Lai Y. (2020). Progress on particulate matter filtration technology: Basic concepts, advanced materials, and performances. Nanoscale.

[38] Chen X., Yang L., Guo J., Xu S., Di J., Zhuang J. (2022). Interactive removal of bacterial and viral particles during transport through low-cost filtering materials. Front. Microbiol..

[39] Zhou J., Hu Z., Zabihi F., Chen Z., Zhu M. (2020). Progress and Perspective of Antiviral Protective Material. Adv. Fiber Mater..

[40] Magee M., Lewis C., Noffs G., Reece H., Chan J.C.S., Zaga C.J., Paynter C., Birchall O., Azocar S.R., Ediriweera A. (2020). Effects of face masks on acoustic analysis and speech perception: Implications for peri-pandemic protocols. J. Acoust. Soc. Am..

[41] Konda A., Prakash A., Moss G.A., Schmoldt M., Grant G.D., Guha S. (2020). Aerosol Filtration Efficiency of Common Fabrics Used in Respiratory Cloth Masks. Acs Nano.

[42] Howard J., Huang A., Li Z.Y., Tufekci Z., Zdimal V., van der Westhuizen H.M., von Delft A., Price A., Fridman L., Tang L.H. (2021). An evidence review of face masks against COVID-19. Proc. Natl. Acad. Sci. USA.

[43] Sharma S.K., Mishra M., Mudgal S.K. (2020). Efficacy of cloth face mask in prevention of novel coronavirus infection transmission: A systematic review and meta-analysis. J. Educ. Health Promot..

[44] Liao M.R., Liu H.Y., Wang X., Hu X.Z., Huang Y.H., Liu X.Q., Brenan K., Mecha J., Nirmalan M., Lu J.R. (2021). A technical review of face mask wearing in preventing respiratory COVID-19 transmission. Curr. Opin. Colloid Interface Sci..

[45] Babaahmadi V., Amid H., Naeimirad M., Ramakrishna S. (2021). Biodegradable and multifunctional surgical face masks: A brief review on demands during COVID-19 pandemic, recent developments, and future perspectives. Sci. Total Environ..

[46] Hao W., Parasch A., Williams S., Li J., Ma H., Burken J., Wang Y. (2020). Filtration performances of non-medical materials as candidates for manufacturing facemasks and respirators. Int. J. Hyg. Environ. Health.

[47] Rashid T.U., Sharmeen S., Biswas S. (2022). Effectiveness of N95 Masks against SARS-CoV-2: Performance Efficiency, Concerns, and Future Directions. Acs Chem. Health Saf..

[48] Weber T.P., Stilianakis N.I. (2008). Inactivation of influenza A viruses in the environment and modes of transmission: A critical review. J. Infect..

[49] Coulliette A.D., Perry K.A., Edwards J.R., Noble-Wang J.A. (2013). Persistence of the 2009 Pandemic Influenza A (H1N1) Virus on N95 Respirators. Appl. Environ. Microbiol..

[50] Hiragond C.B., Kshirsagar A.S., Dhapte V.V., Khanna T., Joshi P., More P.V. (2018). Enhanced anti-microbial response of commercial face mask using colloidal silver nanoparticles. Vacuum.

[51] Gu G.Q., Han C.B., Lu C.X., He C., Jiang T., Gao Z.L., Li C.J., Wang Z.L. (2017). Triboelectric Nanogenerator Enhanced Nanofiber Air Filters for Efficient Particulate Matter Removal. Acs Nano.

[52] Zhong Z.X., Xu Z., Sheng T., Yao J.F., Xing W.H., Wang Y. (2015). Unusual Air Filters with Ultrahigh Efficiency and Antibacterial Functionality Enabled by ZnO Nanorods. Acs Appl. Mater. Interfaces.

[53] Lin Z., Wang Z., Zhang X., Diao D. (2021). Superhydrophobic, photo-sterilize, and reusable mask based on graphene nanosheet-embedded carbon (GNEC) film. Nano Res..

[54] Neisiany R.E., Enayati M.S., Kazemi-beydokhti A., Das O., Ramakrishna S. (2020). Multilayered Bio-Based Electrospun Membranes: A Potential Porous Media for Filtration Applications. Front. Mater..

[55] Yang X., Pu Y., Zhang Y., Liu X., Li J., Yuan D., Ning X. (2020). Multifunctional composite membrane based on BaTiO3@PU/PSA nanofibers for high-efficiency PM2.5 removal. J. Hazard. Mater..

[56] Wang C., Meng N., Babar A.A., Gong X.B., Liu G.H., Wang X.F., Yu J.Y., Ding B. (2022). Highly Transparent Nanofibrous Membranes Used as Transparent Masks for Efficient PM0.3 Removal. Acs Nano.

[57] Barhoum A., Pal K., Rahier H., Uludag H., Kim I.S., Bechelany M. (2019). Nanofibers as new-generation materials: From spinning and nano-spinning fabrication techniques to emerging applications. Appl. Mater. Today.

[58] Huang Z.M., Zhang Y.Z., Kotaki M., Ramakrishna S. (2003). A review on polymer nanofibers by electrospinning and their applications in nanocomposites. Compos. Sci. Technol..

[59] Bian Y., Wang S., Jin D., Wang R., Chen C., Zhang L. (2020). A general anion exchange strategy to transform metal-organic framework embedded nanofibers into high-performance lithium-ion capacitors. Nano Energy.

[60] Nayak R., Padhye R., Kyratzis I., Truong Y.B., Arnold L. (2012). Recent advances in nanofibre fabrication techniques. Text. Res. J..

[61] Dong H., Strawhecker K.E., Snyder J.F., Orlicki J.A., Reiner R.S., Rudie A.W. (2012). Cellulose nanocrystals as a reinforcing material for electrospun poly(methyl methacrylate) fibers: Formation, properties and nanomechanical characterization. Carbohydr. Polym..

[62] Saba N., Paridah M.T., Abdan K., Ibrahim N.A. (2017). A Review on Nano Fibre Technology in Polymer Composites. Pertanika J. Sci. Technol..

[63] Li D., McCann J.T., Xia Y.N. (2005). Use of electrospinning to directly fabricate hollow nanofibers with functionalized inner and outer surfaces. Small.

[64] Ahmed F.E., Lalia B.S., Hashaikeh R. (2015). A review on electrospinning for membrane fabrication: Challenges and applications. Desalination.

[65] Lu T., Deng Y., Cui J., Cao W., Qu Q., Wang Y., Xiong R., Ma W., Lei J., Huang C. (2021). Multifunctional Applications of Blow-Spinning Setaria viridis Structured Fibrous Membranes in Water Purification. Acs Appl. Mater. Interfaces.

[66] Zhou C., Sun Y., Zhang F., Wu Y. (2021). Degradation of Minocycline by the Adsorption-Catalysis Multifunctional PVDF-PVP-TiO2 Membrane: Degradation Kinetics, Photocatalytic Efficiency, and Toxicity of Products. Int. J. Environ. Res. Public Health.

[67] Persano L., Camposeo A., Tekmen C., Pisignano D. (2013). Industrial Upscaling of Electrospinning and Applications of Polymer Nanofibers: A Review. Macromol. Mater. Eng..

[68] Han W., Rao D., Gao H., Yang X., Fan H., Li C., Dong L., Meng H. (2022). Green-solvent-processable biodegradable poly(lactic acid) nanofibrous membranes with bead-on-string structure for effective air filtration: “Kill two birds with one stone”. Nano Energy.

[69] Parham S., Kharazi A.Z., Bakhsheshi-Rad H.R., Ghayour H., Ismail A.F., Nur H., Berto F. (2020). Electrospun Nano-Fibers for Biomedical and Tissue Engineering Applications: A Comprehensive Review. Materials.

[70] Yoo H.S., Kim T.G., Park T.G. (2009). Surface-functionalized electrospun nanofibers for tissue engineering and drug delivery. Adv. Drug Deliv. Rev..

[71] Bian Y., Zhang L., Chen C. (2018). Experimental and modeling study of pressure drop across electrospun nanofiber air filters. Build. Environ..

[72] Bian Y., Wang S., Zhang L., Chen C. (2020). Influence of fiber diameter, filter thickness, and packing density on PM2.5 removal efficiency of electrospun nanofiber air filters for indoor applications. Build. Environ..

[73] Shanmugam V., Babu K., Garrison T.F., Capezza A.J., Olsson R.T., Ramakrishna S., Hedenqvist M.S., Singha S., Bartoli M., Giorcelli M. (2021). Potential natural polymer-based nanofibres for the development of facemasks in countering viral outbreaks. J. Appl. Polym. Sci..

[74] Huang W.W., Ling S.J., Li C.M., Omenetto F.G., Kaplan D.L. (2018). Silkworm silk-based materials and devices generated using bio-nanotechnology. Chem. Soc. Rev..

[75] Rockwood D.N., Preda R.C., Yucel T., Wang X.Q., Lovett M.L., Kaplan D.L. (2011). Materials fabrication from Bombyx mori silk fibroin. Nat. Protoc..

[76] Sun W., Gregory D.A., Tomeh M.A., Zhao X. (2021). Silk Fibroin as a Functional Biomaterial for Tissue Engineering. Int. J. Mol. Sci..

[77] Woltje M., Kolbel A., Aibibu D., Cherif C. (2021). A Fast and Reliable Process to Fabricate Regenerated Silk Fibroin Solution from Degummed Silk in 4 Hours. Int. J. Mol. Sci..

[78] Melke J., Midha S., Ghosh S., Ito K., Hofmann S. (2016). Silk fibroin as biomaterial for bone tissue engineering. Acta Biomater..

[79] Gao X.C., Gou J., Zhang L., Duan S.S., Li C.Z. (2018). A silk fibroin based green nano-filter for air filtration. Rsc Adv..

[80] Selvaraj S., Fathima N.N. (2017). Fenugreek Incorporated Silk Fibroin Nanofibers-A Potential Antioxidant Scaffold for Enhanced Wound Healing. Acs Appl. Mater. Interfaces.

[81] Wang Z.K., Cui Y.H., Feng Y.H., Guan L., Dong M.D., Liu Z., Liu L. (2021). A versatile Silk Fibroin based filtration membrane with enhanced mechanical property, disinfection and biodegradability. Chem. Eng. J..

[82] Wang C., Wu S., Jian M., Xie J., Xu L., Yang X., Zheng Q., Zhang Y. (2016). Silk nanofibers as high efficient and lightweight air filter. Nano Res..

[83] Bian Y., Wang R.T., Ting S.H., Chen C., Zhang L. (2018). Electrospun SF/PVA Nanofiber Filters for Highly Efficient PM2.5 Capture. IEEE Trans. Nanotechnol..

[84] Siskova A.O., Mosnackova K., Hruza J., Frajova J., Opalek A., Buckova M., Kozics K., Peer P., Andicsova A.E. (2021). Electrospun Poly(ethylene Terephthalate)/Silk Fibroin Composite for Filtration Application. Polymers.

[85] Wang Z., Song X., Cui Y., Cheng K., Tian X., Dong M., Liu L. (2021). Silk fibroin H-fibroin/poly(epsilon-caprolactone) core-shell nanofibers with enhanced mechanical property and long-term drug release. J. Colloid Interface Sci..

[86] Soo X.Y.D., Wang S., Yeo C.C.J., Li J., Ni X.P., Jiang L., Xue K., Li Z., Fei X., Zhu Q. (2022). Polylactic acid face masks: Are these the sustainable solutions in times of COVID-19 pandemic?. Sci. Total Environ..

[87] Mei L., Ren Y.M., Gu Y.C., Li X.L., Wang C., Du Y., Fan R.R., Gao X., Chen H.F., Tong A.P. (2018). Strengthened and Thermally Resistant Poly(lactic acid)-Based Composite Nanofibers Prepared via Easy Stereocomplexation with Antibacterial Effects. Acs Appl. Mater. Interfaces.

[88] Lin J., Ding B., Yu J., Hsieh Y. (2010). Direct Fabrication of Highly Nanoporous Polystyrene Fibers via Electrospinning. Acs Appl. Mater. Interfaces.

[89] Zuo W.W., Zhu M.F., Yang W., Yu H., Chen Y.M., Zhang Y. (2005). Experimental study on relationship between jet instability and formation of beaded fibers during electrospinning. Polym. Eng. Sci..

[90] Fong H., Chun I., Reneker D.H. (1999). Beaded nanofibers formed during electrospinning. Polymer.

[91] Gupta P., Elkins C., Long T.E., Wilkes G.L. (2005). Electrospinning of linear homopolymers of poly(methyl methacrylate): Exploring relationships between fiber formation, viscosity, molecular weight and concentration in a good solvent. Polymer.

[92] Wang Z., Zhao C.C., Pan Z.J. (2015). Porous bead-on-string poly(lactic acid) fibrous membranes for air filtration. J. Colloid Interface Sci..

[93] Wang L., Gao Y.F., Xiong J.P., Shao W.L., Cui C., Sun N., Zhang Y.T., Chang S.Z., Han P.J., Liu F. (2022). Biodegradable and high-performance multiscale structured nanofiber membrane as mask filter media via poly(lactic acid) electrospinning. J. Colloid Interface Sci..

[94] Raafat D., Leib N., Wilmes M., Francois P., Schrenzel J., Sahl H.G. (2017). Development of in vitro resistance to chitosan is related to changes in cell envelope structure of Staphylococcus aureus. Carbohydr. Polym..

[95] Sargazi G., Afzali D., Mostafavi A., Ebrahimipour S.Y. (2018). Synthesis of CS/PVA Biodegradable Composite Nanofibers as a Microporous Material with Well Controllable Procedure Through Electrospinning. J. Polym. Environ..

[96] Zhang B., Zhang Z.G., Yan X., Wang X.X., Zhao H., Guo J., Feng J.Y., Long Y.Z. (2017). Chitosan nanostructures by in situ electrospinning for high-efficiency PM2.5 capture. Nanoscale.

[97] Dilamian M., Montazer M., Masoumi J. (2013). Antimicrobial electrospun membranes of chitosan/poly(ethylene oxide) incorporating poly(hexamethylene biguanide) hydrochloride. Carbohydr. Polym..

[98] Wang C., Fan J., Xu R., Zhang L., Zhong S., Wang W., Yu D. (2019). Quaternary ammonium chitosan/polyvinyl alcohol composites prepared by electrospinning with high antibacterial properties and filtration efficiency. J. Mater. Sci..

[99] Pan W., Wang J.P., Sun X.B., Wang X.X., Jiang J.Y., Zhang Z.G., Li P., Qu C.H., Long Y.Z., Yu G.F. (2021). Ultra uniform metal-organic framework-5 loading along electrospun chitosan/polyethylene oxide membrane fibers for efficient PM2.5 removal. J. Clean. Prod..

[100] Li H., Wang Z., Zhang H.Y., Pan Z.J. (2018). Nanoporous PLA/(Chitosan Nanoparticle) Composite Fibrous Membranes with Excellent Air Filtration and Antibacterial Performance. Polymers.

[101] Croisier F., Jerome C. (2013). Chitosan-based biomaterials for tissue engineering. Eur. Polym. J..

[102] Sharma S., Batra S., Holban A.-M., Grumezescu A.M. (2019). Chapter 5—Recent advances of chitosan composites in artificial skin: The next era for potential biomedical application. Materials for Biomedical Engineering.

[103] Ilyas R.A., Aisyah H.A., Nordin A., Ngadi N., Zuhri M.Y.M., Asyraf M.R.M., Sapuan S.M., Zainudin E.S., Sharma S., Abral H. (2022). Natural-Fiber-Reinforced Chitosan, Chitosan Blends and Their Nanocomposites for Various Advanced Applications. Polymers.

[104] Min B.M., Lee S.W., Lim J.N., You Y., Lee T.S., Kang P.H., Park W.H. (2004). Chitin and chitosan nanofibers: Electrospinning of chitin and deacetylation of chitin nanofibers. Polymer.

[105] Peng B.L., Yao Z.L., Wang X.C., Crombeen M., Sweeney D.G., Tam K.C. (2020). Cellulose-based materials in wastewater treatment of petroleum industry. Green Energy Environ..

[106] Alavi M. (2019). Modifications of microcrystalline cellulose (MCC), nanofibrillated cellulose (NFC), and nanocrystalline cellulose (NCC) for antimicrobial and wound healing applications. E-Polymers.

[107] Stanislas T.T., Tendo J.F., Ojo E.B., Ngasoh O.F., Onwualu P.A., Njeugna E., Savastano H. (2022). Production and Characterization of Pulp and Nanofibrillated Cellulose from Selected Tropical Plants. J. Nat. Fibers.

[108] Jiang F., Hsieh Y.-L. (2015). Cellulose nanocrystal isolation from tomato peels and assembled nanofibers. Carbohydr. Polym..

[109] Ma Z.W., Kotaki M., Ramakrishna S. (2005). Electrospun cellulose nanofiber as affinity membrane. J. Membr. Sci..

[110] Balgis R., Murata H., Goi Y., Ogi T., Okuyama K., Bao L. (2017). Synthesis of Dual-Size Cellulose-Polyvinylpyrrolidone Nanofiber Composites via One-Step Electrospinning Method for High-Performance Air Filter. Langmuir.

[111] Sun B., Lin J., Liu M., Li W., Yang L., Zhang L., Chen C., Sun D. (2022). In Situ Biosynthesis of Biodegradable Functional Bacterial Cellulose for High-Efficiency Particulate Air Filtration. Acs Sustain. Chem. Eng..

[112] Hardian R., Alammar A., Holtzl T., Szekely G. (2022). Fabrication of sustainable organic solvent nanofiltration membranes using cellulose-chitosan biopolymer blends. J. Membr. Sci..

[113] Gianazza E., Viglienghi V., Righetti P.G., Salamini F., Soave C. (1977). Amino-Acid Composition of Zein Molecular Components. Phytochemistry.

[114] Khansari S., Sinha-Ray S., Yarin A.L., Pourdeyhimi B. (2013). Biopolymer-Based Nanofiber Mats and Their Mechanical Characterization. Ind. Eng. Chem. Res..

[115] Park J.H., Park S.M., Kim Y.H., Oh W., Lee G.W., Karim M.R., Park J.H., Yeum J.H. (2013). Effect of montmorillonite on wettability and microstructure properties of zein/montmorillonite nanocomposite nanofiber mats. J. Compos. Mater..

[116] Fan X., Wang Y., Zhong W.H., Pan S.Y. (2019). Hierarchically Structured All-biomass Air Filters with High Filtration Efficiency and Low Air Pressure Drop Based on Pickering Emulsion. Acs Appl. Mater. Interfaces.

[117] Souzandeh H., Scudiero L., Wang Y., Zhong W.-H. (2017). A Disposable Multi-Functional Air Filter: Paper Towel/Protein Nanofibers with Gradient Porous Structures for Capturing Pollutants of Broad Species and Sizes. Acs Sustain. Chem. Eng..

[118] Tian H.F., Fu X.W., Zheng M., Wang Y., Li Y.C., Xiang A.M., Zhong W.H. (2018). Natural polypeptides treat pollution complex: Moisture-resistant multi-functional protein nanofabrics for sustainable air filtration. Nano Res..

[119] Yu X., Li C.M., Tian H.F., Yuan L., Xiang A.M., Li J.L., Wang C.Y., Rajulu A.V. (2020). Hydrophobic cross-linked zein-based nanofibers with efficient air filtration and improved moisture stability. Chem. Eng. J..

[120] Hu J., Xiong Z.J., Liu Y.Q., Lin J.Y. (2022). A biodegradable composite filter made from electrospun zein fibers underlaid on the cellulose paper towel. Int. J. Biol. Macromol..

[121] Fan X., Wang Y., Zheng M., Dunne F., Liu T., Fu X.W., Kong L., Pan S.Y., Zhong W.H. (2018). Morphology engineering of protein fabrics for advanced and sustainable filtration. J. Mater. Chem. A.

[122] Liu J.J., Dunne F.O., Fan X., Fu X.W., Zhong W.H. (2019). A protein-functionalized microfiber/protein nanofiber Bi-layered air filter with synergistically enhanced filtration performance by a viable method. Sep. Purif. Technol..

[123] Fu X.W., Liu J.J., Ding C.F., Lin S.N., Zhong W.H. (2021). Building bimodal structures by a wettability difference-driven strategy for high-performance protein air-filters. J. Hazard. Mater..

[124] Mosher C.Z., Brudnicki P.A.P., Gong Z.X., Childs H.R., Lee S.W., Antrobus R.M., Fang E.C., Schiros T.N., Lu H.H. (2021). Green electrospinning for biomaterials and biofabrication. Biofabrication.

[125] Castilla-Casadiego D.A., Maldonado M., Sundaram P., Almodovar J. (2016). "Green" electrospinning of a collagen/hydroxyapatite composite nanofibrous scaffold. Mrs Commun..

[126] Zakrzewska A., Bayan M.A.H., Nakielski P., Petronella F., De Sio L., Pierini F. (2022). Nanotechnology Transition Roadmap toward Multifunctional Stimuli-Responsive Face Masks. Acs Appl. Mater. Interfaces.

[127] De Sio L., Ding B., Focsan M., Kogermann K., Pascoal-Faria P., Petronela F., Mitchell G., Zussman E., Pierini F. (2021). Personalized Reusable Face Masks with Smart Nano-Assisted Destruction of Pathogens for COVID-19: A Visionary Road. Chem.-A Eur. J..

[128] Le T.T., Curry E.J., Vinikoor T., Das R., Liu Y., Sheets D., Tran K.T.M., Hawxhurst C.J., Stevens J.F., Hancock J.N. (2022). Piezoelectric Nanofiber Membrane for Reusable, Stable, and Highly Functional Face Mask Filter with Long-Term Biodegradability. Adv. Funct. Mater..

